# Development and Evaluation of Vero Cell-Derived Master Donor Viruses for Influenza Pandemic Preparedness

**DOI:** 10.3390/vaccines8040626

**Published:** 2020-10-25

**Authors:** Po-Ling Chen, Tsai-Teng Tzeng, Alan Yung-Chih Hu, Lily Hui-Ching Wang, Min-Shi Lee

**Affiliations:** 1 National Institution of Infectious Diseases and Vaccinology, National Health Research Institutes (NHRI), Zhunan, Miaoli 35053, Taiwan; letitia@nhri.edu.tw (P.-L.C.); jtzeng@nhri.edu.tw (T.-T.T.); alanhu@nhri.edu.tw (A.Y.-C.H.); 2 Institute of Molecular and Cellular Biology, National Tsing Hua University, Hsinchu 300044, Taiwan; lilywang@life.nthu.edu.tw

**Keywords:** pandemic preparedness, Vero cell-derived influenza vaccine, master donor virus

## Abstract

The embryonated egg-based platform currently produces the majority of seasonal influenza vaccines by employing a well-developed master donor virus (MDV, A/PR/8/34 (PR8)) to generate high-growth reassortants (HGRs) for A/H1N1 and A/H3N2 subtypes. Although the egg-based platform can supply enough seasonal influenza vaccines, it cannot meet surging demands during influenza pandemics. Therefore, multi-purpose platforms are desirable for pandemic preparedness. The Vero cell-based production platform is widely used for human vaccines and could be a potential multi-purpose platform for pandemic influenza vaccines. However, many wild-type and egg-derived influenza viruses cannot grow efficiently in Vero cells. Therefore, it is critical to develop Vero cell-derived high-growth MDVs for pandemic preparedness. In this study, we evaluated two in-house MDVs (Vero-15 and VB5) and two external MDVs (PR8 and PR8-HY) to generate Vero cell-derived HGRs for five avian influenza viruses (AIVs) with pandemic potentials (H5N1 clade 2.3.4, H5N1 clade 2.3.2.1, American-lineage H5N2, H7N9 first wave and H7N9 fifth wave). Overall, no single MDV could generate HGRs for all five AIVs, but this goal could be achieved by employing two in-house MDVs (vB5 and Vero-15). In immunization studies, mice received two doses of Vero cell-derived inactivated H5N1 and H7N9 whole virus antigens adjuvanted with alum and developed robust antibody responses.

## 1. Introduction

Vaccine supply for unpredictable pandemics is a challenge. For the supply of influenza vaccines, there is a conflict between the demand of seasonal and pandemic vaccines. Currently, egg-based, cell-based, and recombinant protein-based platforms are used for influenza vaccines. The egg-based platform is widely used and could provide enough seasonal influenza vaccines, but it is hard to meet a surging demand during pandemics because of the long production time [1]. The other two platforms could produce influenza vaccines in a short time, which accelerates vaccine production and increases vaccine supply during pandemics [2]. However, these two platforms are used in a limited number of countries with low market share of seasonal vaccines (about 10–15% in the USA) [3]. Since production of seasonal influenza vaccines is over demand at the moment, it is not practical to build additional facilities just for pandemic vaccine production. Therefore, multi-purpose platforms are desirable for pandemic preparedness [4].

A multi-purpose platform could be used to produce other human vaccines and develop pandemic influenza vaccines for clinical trials during influenza pre-pandemic phases. Once pandemics hit, the multi-purpose platform could be converted to produce pandemic influenza vaccines to speed up and increase vaccine supply. One potential multi-purpose production platform is Vero cell culture system. Vero cells have been widely used for production of human viral vaccines, such as rotavirus, Japanese encephalitis virus, rabies virus, poliovirus, and other emerging viruses [5]. Therefore, the well-established Vero cell-based facilities could be used for production of pandemic influenza vaccines during influenza pandemics [4].

**Figure 1:**
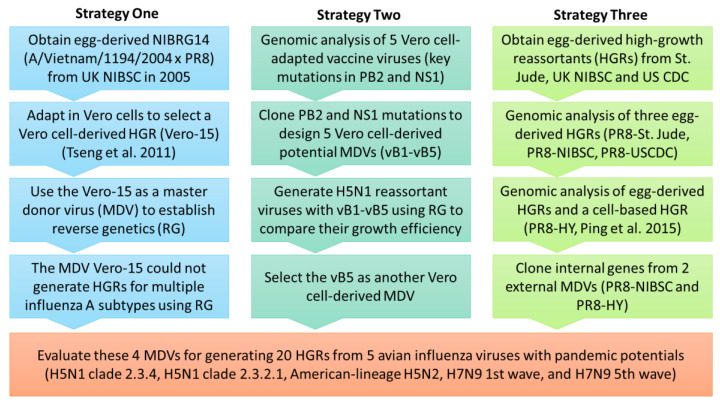
The study flowchart.

Most wild-type influenza viruses and egg-derived high-growth reassortants (HGRs, >10^7.5^ TCID_50_/mL) may not grow efficiently in Vero cells and need adaptations [6], which is time-consuming and not feasible for emergency response. Since the 2017–2018 season, the World Health Organization (WHO) has provided MDCK cell-derived wild-type viruses to be used as seasonal influenza CVVs for MDCK cell-based production. The growth of MDCK cell-derived CVVs in Vero cells still need to be verified. To increase production efficiency, it is desirable to develop high-growth influenza viruses as master donor viruses (MDVs) for the Vero cell-based platform. Our laboratory previously adapted an egg-derived high-growth H5N1 clade 1 CVV in Vero cells (Vero-15) [6] (Figure 1, strategy one) and applied Vero-15 as an MDV to generate high-growth reassortant viruses for avian influenza viruses (AIVs). Our previous data shows that Vero-15 could generate HGRs of H5N1 clade 2 viruses but not American-lineage H5N2 viruses [7]. Therefore, it is desirable to develop other MDVs to establish the Vero cell-based production platform for multiple AIVs.

A/Puerto Rico/8/34 (H1N1), PR8, is an egg-derived high-growth MDV for egg-based production platform, and widely used to generate CVVs. Our goal is to modify the PR8 MDV to develop high-growth MDVs for Vero cell-based production platform. Overall, we evaluated four MDVs (two in-house MDVs and two external MDVs, PR8 and PR8-HY [8]) to generate Vero cell-derived HGRs for five AIVs with pandemic potentials, including H5N1 clade 2.3.4, H5N1 clade 2.3.2.1, American-lineage H5N2, H7N9 first wave, and H7N9 fifth wave (Figure 1). Moreover, mice immunization studies were conducted to evaluate immunogenicity of inactivated whole virus antigens from Vero cell-derived H5N1 and H7N9 viruses.

## 2. Materials and Methods

### 2.1. Cells and Viruses

Vero cells were obtained from the American Type Culture Collection (ATCC CCL-81, Manassas, VA, USA) and cultivated in M199 medium (Thermo Fisher Scientific, Waltham, MA, USA) containing 5% fetal bovine serum (FBS) (Moregate, QLD, Australia) or serum-free medium (VP-SFM, Thermo Fisher Scientific, MA, USA). Several egg-derived CVVs, NIBRG-14 (A/Vietnam/1194/2004 (H5N1) × PR8) and NIBRG-268 (A/Anhui/1/2013 (first H7N9 wave) × PR8) were provided by National Institute for Biological Standards and Control (NIBSC), Herts, UK, and IDCDC-RG6 (A/Anhui/1/2005 (H5N1) × PR8), IDCDC-RG30 (A/Hubei/1/2010 (H5N1) × PR8), IDCDC-RG32A (A/Shanghai/2/2013 (1st H7N9 wave) × PR8) were provided by the Centers for Disease Control and Prevention (CDC), Atlanta, GA, USA. Wild-type virus, A/CK/YL/0502/2012 (American-lineage H5N2 virus, R3) was provided by Animal Health Research Institute, Taiwan [7].

### 2.2. Plasmid Construction

Viral RNAs were extracted using VIOGENE^®^ Viral RNA Extraction Miniprep System (Diagnostic Technology, NSW, Australia), and reverse transcribed by HiScript I Reverse Transcriptase kit (Bionovas, ON, Canada) with Uni12 primer. Influenza virus gene segments were amplified by PFU Ultra II Fusion HS DNA Polymerase (Agilent, Santa Clara, CA, USA) with universal primers developed by Hoffmann et al., 2001. The amplified genes were cloned to vector pHW2000 to express both viral RNAs and proteins [9]. The six internal genes from NIBRG-14 were from the egg-adapted high growth control master donor virus, PR8. The PB2-S360Y mutation was from Vero cell-adapted NIGRG-14, Vero-15 [6]. The Vero cell-derived NS genes were from Vero cell-adapted viruses, Vero-15, Vero-16, RG6-Vero, RG30-Vero, and RG32A-Vero (Table 1). The six internal genes of PR8-HY were generated by modifying internal genes of PR8-Saint Jude Children’s Research Hospital based on the sequences published by Ping et al., 2015 [8] (Table S2). The HA and NA genes of RG6, RG30, R3, and RG268 were cloned from viruses and the HA and NA of RG56B were synthesized based on the sequence of IDCDC-RG56B (A/Hong Kong/125/2017 (fifth H7N9 wave) × PR8). All primers used in this study are listed in Table S1.

### 2.3. Generation of Reassortant Viruses Using Electroporation

Eight expression plasmids carrying genes of influenza virus and suspended Vero cells (5 × 10^6^ cells/reaction) were mixed and electroporated to generate reassortant viruses. The condition of electroporation was two pulses in a 4-mm electroporation cuvette. After electroporation, the transfected cells were transferred to 6-well plates and cultured with M199 medium with 5% FBS at 37 °C for 16 h. In the second day, the medium was replaced with M199 medium with 1–2 μg/mL TPCK-trypsin, and then incubated at 35 °C for 4 days.

### 2.4. Virus Titration

Viruses were titrated using a haemagglutination assay (HA assay), 50% Tissue Culture Infective Dose (TCID_50_) assay, and plaque assay. The HA assay was conducted in 96-well V-shaped and U-shaped microplates with 0.5% turkey red blood cells (TKRBCs) and 0.25% TKRBCs, respectively [10,11]. The TCID_50_ assay and plaque assay was conducted with Vero cells [10].

### 2.5. Growth Curve

Virus growth was evaluated in T75 flasks and spinner flasks in serum-free culture system. Vero cells were cultivated with VP-SFM medium and incubated at 37 °C. After virus infection, the medium was replaced with VP-SFM containing 1 μg/mL of TPCK-trypsin and the cells were incubated at 35 °C. TPCK-trypsin was supplied every day, and samples were collected every day for measuring the virus growth.

### 2.6. Antigen Preparation

Harvested viruses were inactivated with 0.01% formaldehyde at 37 °C for overnight. The inactivated viruses were purified by using ultracentrifuge (100,000× *g*, 4 °C, 1.5 h with 20% sucrose as a cushion. Protein content was measured by Lowry assay (Pierce™ Modified Lowry protein assay kit, Thermo Fisher Scientific) and HA contents were measured by using single radial immunodiffusion (SRID) [12] with the WHO standard reagents. If the WHO standard reagents were not available, HA content was measured by using densitometry of SDS-PAGE [13].

### 2.7. Mouse Immunization

Six-week-old female BALB/c mice were used for immunization studies (six mice/group). Mice were immunized with 0.05 or 0.2 μg of antigen with 300 μg of Al(OH)_3_ and boosted with the same dosages at the 14th day after the first immunization. The blood samples were taken the day prior to immunization, and at the 14th and 28th day after the first immunization. All mice were sacrificed on the 28th day after the first immunization. The haemagglutinin inhibition assay (HI assay) and microneutralisation assay (MN assay) were based on WHO standard procedures [10].

### 2.8. Viral RNA Expression

Cells were inoculated with viruses (10 MOI) in medium with 2 μg/mL TPCK-trypsin at 35 °C for 1 h. After inoculation, the virus was removed, and cells were washed with 1X dPBS. Fresh medium (with TPCK-trypsin) was added to the cells and incubated at 35 °C. The incubation was stopped at 0, 1, 2, 3, 4, 8, 12, 20, and 28 h post-infection (hpi). The cells were collected and stored at −80 °C. The viral RNA expression was measured using real-time polymerase chain reaction (Q-PCR) [14].

### 2.9. Real-Time Polymerase Chain Reaction (Q-PCR)

Total RNAs from virus-infected cells were extracted by using VIOGENE FFPE miTotal™ Extraction Miniprep System. The viral RNAs were reverse transcribed to cDNA with Uni12 primer, and the mRNAs were transcribed with oligo dT primer. Real-time PCR was conducted with Applied Biosystems^®^ 96-well Real-Time PCR systems and Applied Biosystems Power SYBR^®^ Green PCR master mix (Cat No. 4367659, Applied Biosystems, Foster City, CA, USA). Primers used for qPCR are listed in Table S1.

### 2.10. Ethics Statement

Animal experiments were conducted following the guidelines of Institutional Committee on Animal Care and Use based on the Institutional Animal Care Committee Guidebook published by the US Office of Laboratory Animal Welfare under the supervision of Animal Centre in National Health Research Institutes (Protocol No: NHRI-IACUC-107106-A).

## 3. Results

### 3.1. Genetic Markers Related to High-Growth Influenza Viruses in Vero Cells

To identify genetic markers related to high-growth influenza viruses in Vero cells, genome sequences of five Vero cell-derived viruses in our laboratory were analyzed and compared with PR8-NIBSC (Table 1). Two molecular markers PB2-S360Y and mutated NS1 were identified in most of Vero cell-derived high-growth viruses. The PB2-S360Y mutation has been confirmed to enhance the activity of viral RNA-dependent RNA polymerase in MDCK cells [15], so it may enhance the virus growth in Vero cells. Mutated NS1 was found in all five Vero cell-derived vaccine viruses in our laboratory. Those mutations on NS1 were mainly located on effector domain, but different Vero cell-adapted viruses had different mutations.

### 3.2. Generation of Reassortant Viruses Using Candidate Master Donor Viruses

**Figure 2:**
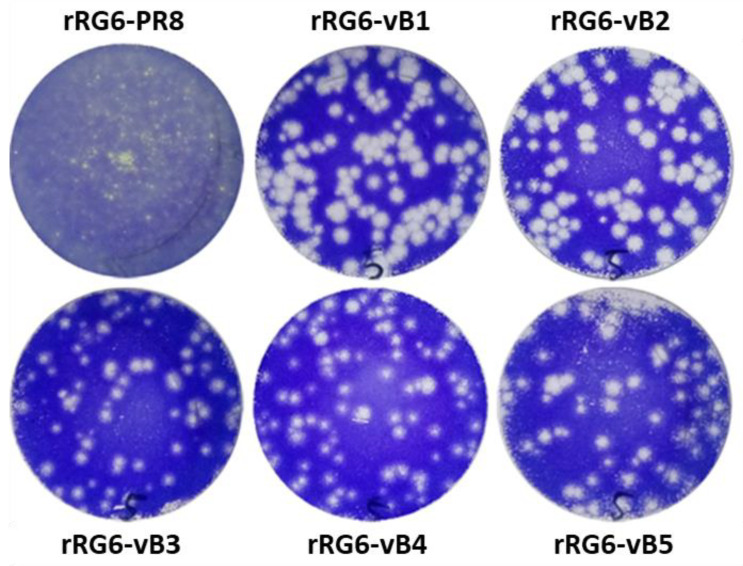
The plaque formation of reassortant viruses in Vero cells.

We designed five potential MDVs (vB1–vB5) carrying PB2-S360Y and mutated NS1 genes and the other genes from an IDCDC-RG6 to generate five reassortant viruses (rRG6-vB1 to rRG6-vB5) and a control virus, rRG6 (Table 2). The rRG6-vB1 to rRG6-vB5 viruses were amplified in Vero cells for two passages, and then virus stocks were generated with 0.001 MOI (multiplicity of infection). rRG6 virus was amplified in MDCK cells because it could not grow efficiently in Vero cells. The HA and virus titers of virus stocks were measured in Vero cells (Table 2). All viruses had similar HA titers (about 128 HAU, Haemagglutination titer) and infectious virus titers (>10^7^ TCID_50_/mL). The plaque formation was analyzed in Vero cells, and it shows that the plaques of rRG6-vB1 to rRG6-vB5 were larger than that of rRG6 (Figure 2).

### 3.3. Growth Property of Reassortant Viruses

**Figure 3:**
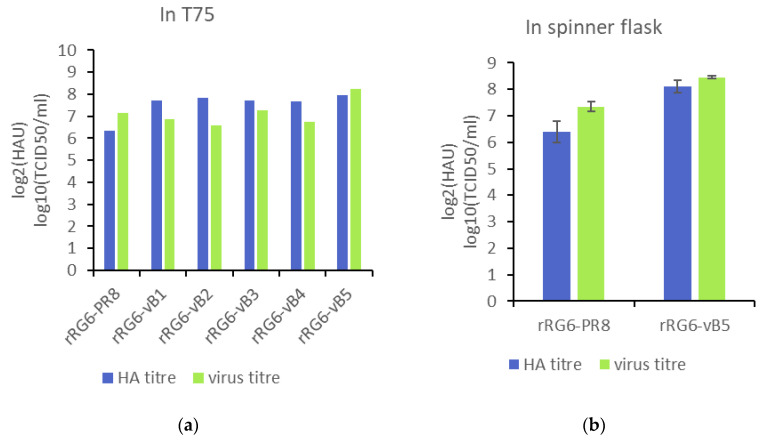
The growth property of reassortant viruses in serum-free medium. The multiplicity of infection (MOI) was 0.0001. TPCK-trypsin was supplied, and the samples were collected every day for Tissue Culture Infective Dose (TCID) assay and hemagglutination assay (HA). (**a**) The peak titer of virus growth in T75 flasks. (**b**) The peak titer of virus growth in spinner flasks.

After the generation of reassortant viruses, the growth was measured in a serum-free culture system, which reduces risk of contamination from adventitious agents [16]. In T75 flasks, rRG6 and rRG6-vB1 to rRG6-vB4 viruses had similar peak virus titer (around 10^7^ TCID_50_/mL), except rRG6-vB5, whose virus titer reached 1.78 × 10^8^ TCID_50_/mL (Figure 3). In spinner flasks using microcarrier-based cell cultures, the peak infectious virus titer of rRG6-vB5 (2.8 × 10^8^ TCID_50_/mL) was 10-fold higher than that of rRG6 (2.2 × 10^7^ TCID_50_/mL), and the HA titer of rRG6-vB5 (276 HAU) was three-fold higher than that of rRG6 (84 HAU) (Figure 3). These results indicate that rRG6-vB5 could increase virus growth and HA production in Vero cells.

### 3.4. Evaluation of Multiple MDVs for Generating High-Growth Vaccine Viruses

**Figure 4:**
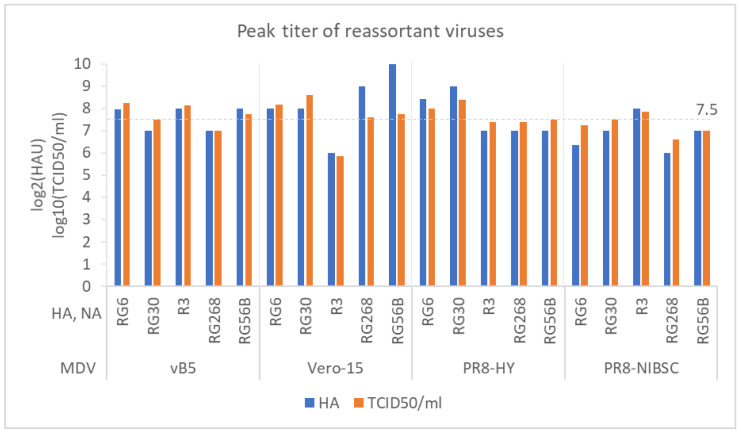
A comparison of virus growth among vB5, Vero-15, PR8-HY, and PR8-NIBSC master donor viruses (MDVs). The HA and NA genes used in this comparison were from RG6, RG30, R3, RG268, and RG56B.

Next, we used MDV vB5 to generate reassortant viruses for other AIVs, including RG30 (A/Hubei/1/2010 (H5N1, clade 2.3.2.1)), R3 (A/CK/YL/0502/2012 (H5N2, American-lineage)), RG268 (A/Anhui/1/2013 (H7N9, first wave)), and RG56B (A/Hong Kong/125/2017 (H7N9, fifth wave)). The growth of reassortant viruses were analyzed in T75 flasks with VP-SFM medium. Peak HA titers of reassortant viruses generated with the MDV vB5 were 128–256 HAU, and peak virus titers were 10^7^–10^8^ TCID_50_/mL. Overall, MDV vB5 could generate high-growth reassortants (HGRs, >10^7.5^ TCID_50_/mL) for RG6, RG30, R3, and RG56B, but not for RG268 (Figure 4).

We also compared MDV vB5 with other MDVs, including Vero-15 [6], PR8-HY [8], and PR8-NIBSC (Table S3). MDV Vero-15 could generate HGRs for RG6, RG30, RG268, and RG56B; MDV PR8-HY could generate HGRs for RG6, RG30, and RG56B; MDV PR8-NIBSC could generate HGRs for RG30 and R3. Overall, these results indicate that multiple MDVs may be a better choice for generating Vero cell-derived HGRs and we could use the two in-house MDVs (Vero-15 and vB5) to generate Vero cell-derived HGRs for all five subtypes of AIVs.

### 3.5. Immunogenicity of Vero Cell-Derived H5N1 and H7N9 Whole Virus Vaccines

Because of the high pandemic potential of avian influenza H5 and H7 viruses (https://www.cdc.gov/flu/pandemic-resources/pdf/cdc-irat-virus-report-asian-avian-H7N9-2017.pdf) [17], we further demonstrated the pilot production of H5N1 and H7N9 vaccines in Vero cells and compared immunogenicity of Vero cell-derived vaccines with that of MDCK cell-derived vaccine candidates in mice. rRG6-vB5 (H5N1 clade 2), rRG268-vB5 (H7N9, the first wave) and rRG56B-vB5 (H7N9, the fifth wave) were selected for mouse immunization study. After purification, HA concentrations of rRG6-vB5 and rRG268-vB5 measured by SRID were 99.05 and 76.89 μg/mL, respectively, and that of rRG56B-vB5 was measured by densitometry as 206.43 μg/mL (Table S4 and Figure S1).

**Figure 5:**
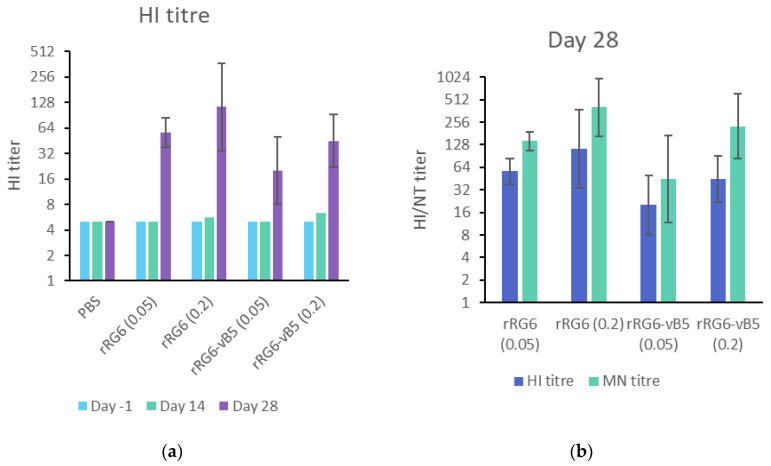
Immunogenicity of multiple RG6 reassortant vaccine viruses in mice. (**a**) Geometric mean titer (GMT) of serum HI antibody in mice receiving different vaccination dosages of RG6 antigens; (**b**) serum HI and neutralization GMT of immunized mouse sera at D28 (14 days after the second immunization). The error bar represents the 95% confidence intervals of GMT.

To evaluate the immunogenicity of Vero and MDCK cell–derived H5N1 antigens (rRG6-vB5 and rRG6-PR8, respectively), mice received two doses of inactivated-whole-virus antigens (0.05 or 0.2 μg) with 300 μg of alum adjuvant two weeks apart. Antibody response rose after the second vaccination, and mice received 0.2 μg of antigens had higher antibody responses than those received 0.05 μg of antigens (Figure 5a). Overall, rRG6-PR8 elicited a slightly higher HI titer than rRG6-vB5 in low (Geometric mean titer, GMT: 56.57 vs. 20) and high (GMT: 113.14 vs. 44.9) dosages without reaching statistical significance (*p* > 0.05). Similar patterns were observed in neutralization antibody responses (Figure 5b).

**Figure 6:**
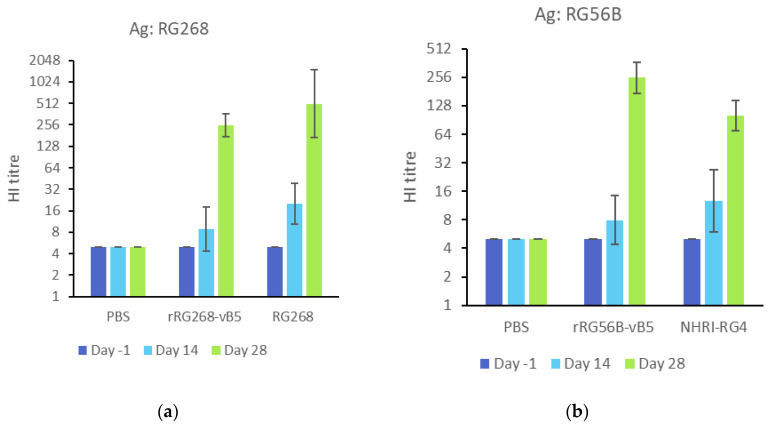

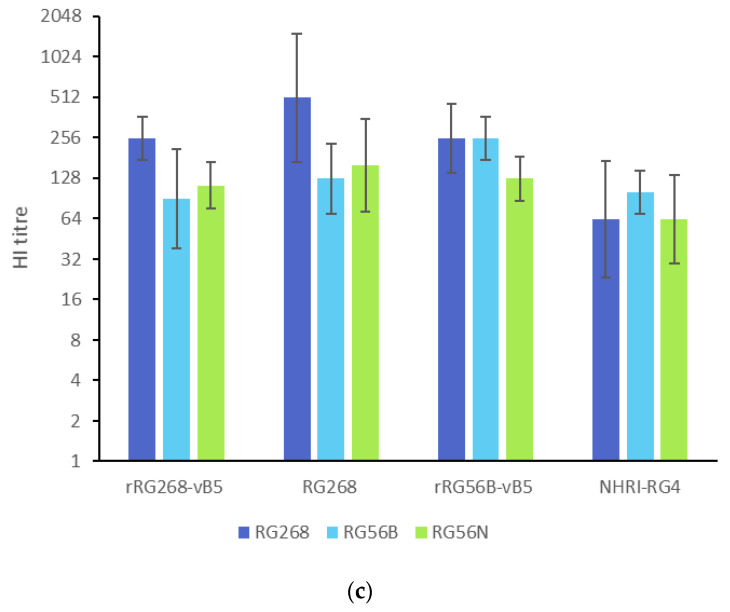
Immunogenicity of rRG268-vB5 and rRG56B-vB5 in mice. (**a**) Sera from rRG268-vB5 immunized mice were used to measure HI titers against homologous antigens (RG268). (**b**) Sera from rRG56B-vB5 immunized mice were used to measure HI titers against homologous antigens (RG56B). NHRI-RG4: MDCK cell-derived CVV carrying HA and NA genes from A/Hong Kong/125/2017. (**c**) Sera from immunized mice were used to measure against heterologous antigens (RG268, RG56B, RG56N: A/Guangdong/17SF003/2016). The error bar represents the 95% of confidence interval.

To evaluate the immunogenicity of Vero cell-derived H7N9 antigens (rRG268-vB5 and rRG56B-vB5), mice received two doses of inactivated-whole-virus antigens (0.2 μg with 300 μg of alum adjuvant) two weeks apart, and another two groups of mice immunized with MDCK cell-derived H7N9 antigens (RG268 and NHRI-RG4, an MDCK cell-derived CVV from NHRI carrying HA and NA genes from A/Hong Kong/125/2017 [18]) as a comparison. In evaluating the homologous antibody response to the first wave H7N9 antigens, the Vero cell-derived antigens (rRG268-vB5) elicited a slightly lower HI antibody response than MDCK cell-derived antigen (RG268) (HI GMT: 253.98 vs. 507.97). By contrast, for the fifth H7N9 antigens, Vero cell-derived antigens (rRG56B-vB5) elicited a slightly higher HI antibody than MDCK cell-derived antigen (NHRI-RG4) (HI GMT: 253.98 vs. 100.79) (Figure 6). Overall, the antibody responses between Vero cell- and MDCK cell-derived antigens did not reach statistical significance (*p* > 0.05). Interestingly, the H7N9 antigens could elicit a stronger homologous antibody response than the H5N1 antigens.

We also analyzed the antibody responses against heterologous antigens from the first and fifth H7N9 waves as shown in Figure 6c. Homologous HI antibody titers were about two- to four-fold higher than heterologous HI antibody titers. Overall, MDCK cell-derived RG268 and Vero cell-derived rRG56B-vB5 had the best homologous and heterologous antibody responses.

### 3.6. Molecular Determinants of Vero Cell-Derived High-Growth MDV vB5

MDV vB5 have two mutated genes, PB2 and NS, compared with MDV PR8-NIBSC. To find the molecular determinant of the growth enhancement, two additional reassortant viruses with either mutated PB2 (rRG6-mPB2) or mutated NS gene (rRG6-NS129) were generated, respectively. Growth efficiency was compared in serum-free culture system. Among these four reassortant viruses, the viruses carrying PB2-S360Y had a significant improvement on both HA and virus titers, and those carrying truncated NS1 had a slight improvement on virus titers (Table 3). These data indicate that the PB2 mutation is the main contributor to increase of HA yield and the double mutations have synergistic effect on virus growth.

**Figure 7:**
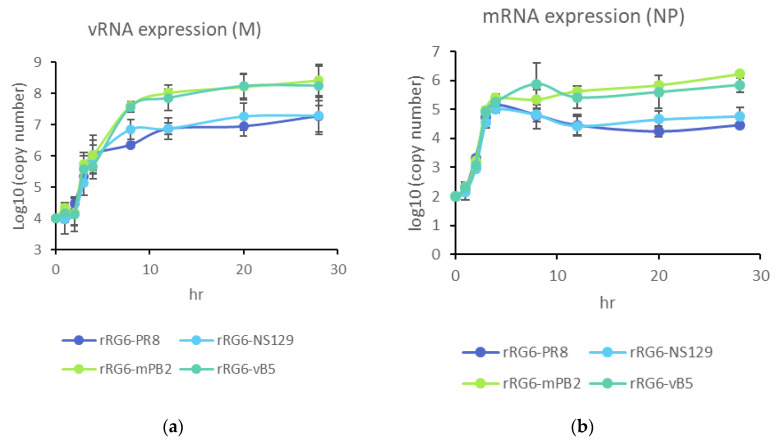
RNA expression in Vero cells after influenza virus infection. Vero cells were infected with 10 MOI. After 0, 1, 2, 3, 4, 8, 12, 20, 28 h post infection, cells were lysed by freeze-thaw action, and extracted total RNA. vRNAs were reverse-transcribed using Uni12 primer (**a**), and mRNAs were measured by using oligo dT primer (**b**).

To further elucidate the effects of PB2-S360Y and truncated NS1 on RdRP activity, we quantify the viral RNA copies in Vero cells and found rRG6-mPB2 and rRG6-vB5 had a higher expression of viral mRNA and vRNA than rRG6-PR8 and rRG6-NS129 (Figure 7). These data indicate that the PB2 mutation could enhance viral RNA expression, which may enhance virus growth in Vero cells.

## 4. Discussion

Although the egg-based production platform is the major supply of seasonal influenza vaccines, the vulnerable chain for the egg supply raises a concern of vaccine supply during pandemics. In addition, low clinical effectiveness of recent egg-derived H3N2 vaccines further demonstrates the urgent need of alternative production platforms, such as cell-based and recombinant protein-based platforms [19]. As discussed previously, the Vero cell-based multifunctional platform is potentially valuable for influenza pandemic preparedness [4], but this platform also needs high-growth MDVs to increase productivity. In this study, we evaluated two in-house MDVs and two external MDVs for generating HGRs with five avian influenza viruses and found that it is hard to generate HGRs for all five avian influenza viruses using one MDVs but this challenge could be overcome by using the two in-house MDVs (Vero-15 and vB5).

The HI antibody responses of H5N1 vaccines were usually low in previous animal and human studies [20,21,22], but some animal studies shows that vaccinated mice with HI titers higher than 40 could survive from lethal challenge [23,24,25]. Moreover, several influenza H5N1 vaccines have been licensed for pre-pandemic use. Immunogenicity of Vero cell-derived whole-virus H5N1 and H7N9 antigens was evaluated in mice in our studies. Based on the homologous HI (GMT 45) and MN (GMT 226) titers in our mouse study, the Vero cell-derived H5N1 whole virus antigens should be further evaluated in ferrets.

The Vero cell-derived H7N9 whole virus antigens could elicit robust homologous HI antibody responses in mice in our study. The Vero cell-derived first wave antigen (rRG268-vB5, HI = 253.98) has a comparable immunogenicity with the MDCK cell-derived RG268 antigen (HI = 507.97) which has been proved to be immunogenic in ferrets [26] and humans [27]. Therefore, it is worthwhile to evaluate Vero cell-derived influenza H7N9 antigens in ferret and human studies.

Based on the risk assessment, influenza H7N9 viruses have a higher risk causing pandemics than influenza H5N1 viruses [28]. There are 21 clinical trials related to H7N9 vaccines (https://www.clinicaltrials.gov/), and 10 of them provide the results [29]. Only three studies used H7N9 vaccine antigens from the fifth wave, and the others used that from the first wave viruses. Without including adjuvants, VLP (NCT01897701), rHA [30] and egg-derived split antigens [31,32,33] all induced HI titers lower than 10, and only cell-derived whole virus antigens could induce HI titers higher than 20 [27]. Among three common adjuvants (alum hydroxyl, AS03, and MF59) used for influenza vaccines, AS03 could strongly improve immunogenicity of split and subunit antigens [33]. Alum and MF59 also show an improvement in immunogenicity but not as strong as AS03 [27,33]. Therefore, it is desirable to evaluate our Vero cell-derived whole virus antigens including different common adjuvants in clinical trials.

To improve growth efficiency of influenza viruses in Vero cells, some techniques have been reported, including using internal genes from adapted viruses [6,34,35,36,37] or introducing Vero cell-adapted mutations [38,39]. In 2015, Ping et al. established random mutation libraries of six internal genes to select MDVs for both egg-based and cell-based platforms from over thousand candidate viruses [8]. The selected MDV (PR8-HY) was compared in this study and could only generate 3 HGRs with TCID50 titers greater than 7.5 log from 5 tested avian influenza viruses. There is a difference between Dr Ping’s study and our study. In the work by Ping et al., (2015), Vero cells were cultivated in MEM with 10% FBS and the medium was replaced with MEM/BSA medium during virus infection, which is an animal component-containing culture system. In our study, we used the serum-free culture system to evaluate the performance of MDVs. The differences in growth efficiency between the serum-containing and serum-free culture systems were also observed when we evaluated the growth efficiency of candidate MDVs (vB1–vB5). The virus titers were about 10^8^ TCID_50_/mL in serum-containing culture system (Figure S2), but were 10^6^–10^7^ TCID_50_/mL in serum-free culture system, except the titer of rRG6-vB5 (Figure 3). Because an animal component-free culture system is suggested to prevent introduction of adventitious agents into vaccine products [16], the results based on serum-free culture system are more relevant to commercial productions.

When influenza viruses grow efficiently in mammalian cells, the pathogenicity of the influenza viruses must be considered. Truncated NS1 has been used to develop live-attenuated influenza vaccines [40,41]. Influenza viruses carrying a truncated NS1 are deficient in the inhibition of IFN signaling pathway and could grow efficiently in IFN-deficient cells, such as Vero cells, but not IFN-expressing cells, like MDCK and human cells [42]. Therefore, we analyzed the growth of rRG6-vB5 in MDCK cells and found a decrease in HA and virus titers (Figure S3), which shows that the MDV vB5 may be a safe MDV for AIVs with pandemic potentials.

## 5. Conclusions

The Vero cell-based vaccine production platform is valuable for influenza pandemic preparedness, but a lack of high-growth MDVs is the drawback. In this study, we evaluated four MDVs for Vero cell-based influenza vaccine production and found that one of the two in-house MDV (vB5 and Vero-15) could generate high-yield vaccine viruses for five H5 and H7N9 viruses. Moreover, Vero cell-derived influenza H7N9 vaccines could elicit a robust HI antibody response in mice and are warrant for further development.

## Figures and Tables

**Table 1:** Genomic analysis of Vero-adapted viruses. Vero-15, Vero-16: two Vero cell-derived high-growth viruses adapted from NIBRG-14. RG6-Vero: Vero-adapted IDCDC-RG6. RG30-Vero: Vero-adapted IDCDC-RG30. RG32A-Vero: Vero-adapted IDCDC-RG32A. NIBRG-14: A/Vietnam/1194/2004 (H5N1) × PR8. IDCDC-RG6: A/Anhui/1/2005 (H5N1) × PR8. IDCDC-RG30: A/Hubei/1/2010 (H5N1) × PR8. IDCDC-RG32A: A/Shanghai/2/2013(H7N9) × PR8.

Virus	Vero-15	Vero-16	RG6-Vero	RG30-Vero	RG32A-Vero
Titer before adaptation	3.16 × 10^5^	3.16 × 10^5^	5.62 × 10^6^	2.51 × 10^6^	7.5 × 10^5^
Titer after adaptation	1.86 × 10^8^	2.9 × 10^8^	4.33 × 10^8^	1 × 10^8^	9.96 × 10^6^
**Protein**	**Amino acid mutations**				
PB2	S360Y	S360Y	R8G	S360F	S360Y
PB1	K197L	K197L	-	-	-
PA	E493G	Y464H	-	-	-
NP	-	N247D S402F S438F	D128N	-	-
M1	-	L233F	-	-	-
M2	-	-	S82N	-	-
NS1	L95P L115P	Truncated NS1 (129aa)	L95P I128T I129T I137T F138L L141P L144P I145T L147P	A23V D120G	Truncated NS1 (127aa)

**Table 2:** Genetic constellation of five reassortant viruses generated using reverse genetics. PR8-NIBSC: A/Puerto Rico/8/34 (H1N1), the current master donor strain using for candidate vaccine viruses from the US Centers for Disease Control and Prevention (CDC) and National Institute for Biological Standards and Control (NIBSC). Amino acid mutations in the NS genes from Vero cell-adapted viruses are shown.

	Control	Reassortant Viruses				
	rRG6 *	rRG6-vB1	rRG6-vB2	rRG6-vB3	rRG6-vB4	rRG6-vB5
HA	A/Anhui/1/2005					
NA						
PB2	PR8-NIBSC	S360Y	S360Y	S360Y	S360Y	S360Y
NS	PR8-NIBSC	L95P, L115P	L95P, I128T, I129T, I137T, F138L, L141P, L144P, I145T, L147P	A23V, D120G	Truncated NS1 (127 a.a.)	Truncated NS1 (129 a.a.)
The other genes	PR8-NIBSC	PR8-NIBSC	PR8-NIBSC	PR8-NIBSC	PR8-NIBSC	PR8-NIBSC
HAU	256	128	128	128	128	128
TCID50/mL	5.62 × 10^7^	3.16 × 10^7^	1.78 × 10^7^	2.04 × 10^7^	4.33 × 10^7^	7.00 × 10^7^

* rRG6 was amplified in MDCK cells.

**Table 3:** The peak titers of reassortant viruses carrying PB2-S360Y or truncated NS1.

	PB2-S360Y Mutation	Truncated NS1	HAU	TCID50/mL
rRG6	no	no	64	5.00 × 10^6^
rRG6-NS129	no	yes	64	1.45 × 10^7^
rRG6-mPB2	yes	no	206	3.98 × 10^7^
rRG6-vB5	yes	yes	249	1.78 × 10^8^

## Supplementary Materials

The following are available online at https://www.mdpi.com/2076-393X/8/4/626/s1, Figure S1. The HA content of purified rRG56B-vB5 was analyzed by densitometry; Figure S2. The growth property of reassortant viruses in M199 medium in six-well plates; Figure S3. The virus growth of rRG6-vB5 in MDCK cells; Table S1. The sequences of the primers used in this study; Table S2. Genetic differences of six internal genes between PR8 from NIBSC, US CDC, St. Jude Children Hospital, and PR8-HY; Table S3. A list of mutations among master donor viruses compared in this study; Table S4. HA antigen yield of influenza H5N1 and H7N9 reassortant vaccine viruses generated using a novel master donor virus.
