# Supplementary material for: Development and Evaluation of Vero Cell-Derived Master Donor Viruses for Influenza Pandemic Preparedness
Source: Vaccines (Basel). 2020 Oct 25;8(4):626. doi: 10.3390/vaccines8040626 (PMC7712210; doi:10.3390/vaccines8040626)
Supplement: Supplementary file 1 [file vaccines-08-00626-s001.pdf]

Table S1. Primers used in this study

Bm-HA-F	TATTCGTCTCAGGGAGCAAAAGCAGGGG	Hoffmann E et al., 2001, Arch Virol
Bm-NS-R	ATATCGTCTCGTATTAGTAGAAACAAGGGT GTTTT	Hoffmann E et al., 2001, Arch Virol
Bm-NA-F	TATTCGTCTCAGGGAGCAAAAGCAGGAG T	Hoffmann E et al., 2001, Arch Virol
Bm-NA-R	ATATCGTCTCGTATTAGTAGAAACAAGGAG TTTTTT	Hoffmann E et al., 2001, Arch Virol
Bm-NS-F	TATTCGTCTCAGGGAGCAAAAGCAGGGT G	Hoffmann E et al., 2001, Arch Virol
Bm-NS-R	ATATCGTCTCGTATTAGTAGAAACAAGGGT GTTTT	Hoffmann E et al., 2001, Arch Virol
Bm-N9uni-F	TATTCGTCTCAGGGAGCAAAAGCAGGGTC	Li J et al., 2014, Emerg Infect Dis.
Bm-N9uni-R	ATATCGTCTCGTATTAGTAGAAACAAGGGT CTTTTT	Li J et al., 2014, Emerg Infect Dis.
Ba-H5-F2	TATTGGTCTCAGGGAGCAAAAGCAGGGG TTCACTCTGTCAAA	This study
Ba-H5-R	ATATGGTCTCGTATTAGTAGAAACAAGGGT GTTTTTAACTAC	This study
H7-820F	GGGAATCCAGAGTGGAGTACAGGTT	This study
H7-1110R	TCCCTGTGCATTCTGGTGTCTG	This study
N9-500F	TGGAATGCATTGGGTGGTCAAGTA	This study
N9-650R	GATTCCTGTGTTCTTAGTATGTTTC	This study
H5-620 F	GATGAGGCAGAGCAGACAAGG	This study
H5-1100R	ATTCCCTGCCATCCTCCCTCTAT	This study
N1-650F	GGCTCTTGCTTTACTGTAATGACTGATG	This study
N1-900R	ATCATTGGGGCGTGGATTGTCT	This study
H5 (H5N2)-670F	CAGAGGTCAATCCCAGAAATAGC	This study
H5 (H5N2)-900R	CATTGTGGAAAGGCATACTGGAA	This study
N2 (H5N2)-600F	GATGCTCGTTGACAGTATAGGTTC	This study
N2 (H5N2)-800R	GTCTCTGCATACACATCTGACATT	This study
PB2-1F	AGCGAAAGCAGGTCAATTATATT	This study
PB2-1R	GGCACATCTCCAATAAAGATGC	This study

PB2-2F	ACATAGTGAGAAGAGCTGCAGTATC	This study
PB2-2R	CCAGTTTCTGATGATCCATTGA	This study
PB2-3F	GATTAATGGTCCTGAATCAGTGTT	This study
PB2-3R	AGTAGAAACAAGGTCGTTTTTAAACT	This study
PB1-1F	AGCGAAAGCAGGCAAACC	This study
PB1-1R	GCCAACTTTGCTTTCTTCTCAT	This study
PB1-2F	TTGAACAATCAGGGTTGCC	This study
PB1-2R	ATGAACAACTGAAGGGCCA	This study
PB1-3F	AATATGATAAACAATGATCTTGGTCC	This study
PB1-3R	AGTAGGAACAAGGCATTTTTTCA	This study
PA-1F	AGCGAAAGCAGGTACTGATCC	This study
PA-1R	GCTTGCCCTCAATGTAGCC	This study
PA-2F	TAGAGCCTATGTGGATGGATTC	This study
PA-2R	CCCTCCTTAGTTCTACACTTGCT	This study
PA-3F	TGCCTTACTTAATGCATCTTGTG	This study
PA-3R	AGTAGAAACAAGGTACTTTTTTGGAC	This study
NP-1F	AGCAAAAGCAGGGTAGATAATCA	This study
NP-1R	TGCAGACCGTGCTAGAAAAGT	This study
NP-2F	CAAGTGAGAGAGAGCCGGG	This study
NP-2R	AGTAGAAACAAGGGTATTTTTCTTTAAT	This study
NS-1F	AGCAAAAGCAGGGTGACAA	This study
NS-1R	AGTAGAAACAAGGGTGTTTTTTATTAT	This study
M1-1F	AGCGAAAGCAGGTAGATATTGA	This study
M1-2R	AGTAGAAACAAGGTAGTTTTTTACTCC	This study
PB2-504V-F	GACCGTTTTTTGAGAGTCCGGGACCAACGAG	This study
PB2-504V-R	CTCGTTGGTCCCGGACTCTCAAAAAACGGTC	This study
PB1-40L-F	GGAACAGGATACACCTTGGATACTGTCAACAGG	This study
PB1-40L-R	CCTGTTGACAGTATCCAAGGTGTATCCTGTTCC	This study
PB1-180W-F	CAATGAACAAAGAAGAAATGTGGATCACAACTCATTTTCAGAG	This study
PB1-180W-R	CTCTGAAAATGAGTTGTGATCCACATTTCT	This study

	TCTTTGTTCATTG	
PA-401K-F	GTGATGAACCAGAATTGAAGTCGCTTGCAAGTTGG	This study
PA-401K-R	CCAACTTGCAAGCGACTTCAATTCTGGTTCATCAC	This study
NS-30P-F	CAAGAACTAGGCGATCCCCCATTCCTTGATCG	This study
NS-30P-R	CGATCAAGGAATGGGGGATCGCCTAGTTCTTG	This study
NS-118K-F	GGCCCTCTTTGTATCAAAATGGACCAGGCGATC	This study
NS-118K-R	GATCGCCTGGTCCATTTTGATACAAAGAGGGCC	This study
NP-116L-F2	CATCCTTTATGACAAAGAAGAATTAAGGCGAATCTGGCG	This study
NP-116L-R2	GCCAGATTCGCCTTAATTCTTCTTTGTCATAAAGGATGAG	This study
M146F	GACCRATCCTGTCACCTCTGAC	Zhu H et al., 2010, Virology
M251R	AGGGCATTYTGGACAAAKCGTCTA	Zhu H et al., 2010, Virology
qNP-514F	CCCAGGATGTGCTCTCTGAT	Khaperskyy DA et al., 2016, PLOS Pathogens
qNP-673R	TTCGTCCATTCTCACCCCTC	Khaperskyy DA et al., 2016, PLOS Pathogens
Beta-actin-F	TGGATCAGCAAGCAGGAGTATG	Zhu H et al., 2010, Virology
beta-actin-R	GCATTTGCGGTGGACGAT	Zhu H et al., 2010, Virology

Table S2. Genetic differences of six internal genes between PR8 from NIBSC, US CDC, St. Jude Children Hospital, and PR8-HY

Gene	Protein	Position	PR8 NIBSC	PR8 USCDC	PR8 St. Jude (PR8-UW)	PR8-HY*
PB2	PB2	105	M	M	I	I
		251	K	K	R	R
		299	K	K	R	R
		360	S	S	Y	Y
		504	V	V	I	V
		702	R	R	K	K
PB1	PB1	40	M	M	M	L
		175	K	K	N	N
		180	G	G	G	W
		205	I	I	M	M
		208	R	R	K	K
		216	G	G	S	S
		563	R	R	I	I
	PB1-F2	59	K	K	R	R
		60	Q	Q	R	R
PA	PA	158	R	R	K	K
		401	R	R	R	K
		550	L	L	I	I
NP	NP	116	I	I	I	L
		353	V	V	L	L
		425	V	V	I	I
		430	T	T	N	N
M	M2	27	A	A	T	T
		39	I	I	T	T
NS	NS1	30	A	A	A	P
		55	E	E	K	K
		101	E	E	D	D
		118	R	R	R	K
	NS2 (NEP)	89	V	V	I	I

*: Ping J, 2015, Nature communication

Table S3. A list of mutations among master donor viruses compared in this study.

	Master donor viruses			
Protein	PR8-NIBSC	vB5	Vero-15 (V15)	PR8-HY (HY)
PB2	-	S360Y	S360Y	M105I<br>K251R<br>K299R<br>S360Y<br>R702K
PB1	-	-	K197L	M40L<br>K175N<br>G180W<br>I205M<br>R208K<br>G216S<br>R563I
PB1-F2	-	-	-	K59R<br>Q60R
PA	-	-	E493G	R158K<br>R401K<br>L550I
NP	-	-	-	I116L<br>V353L<br>V425I<br>T430N
M1	-	-	-	-
M2	-	-	-	A27T<br>I39T
NS1	-	Truncated<br>NS1<br>(129 a.a.)	L95P<br>L115P	A30P<br>E55K<br>E101D<br>R118K
NEP	-	-	-	V89I

Table S4. **HA antigen yield of influenza H5N1 and H7N9 reassortant vaccine viruses generated using MDV vB5.**

	rRG6-vB5	rRG268-vB5	rRG56B-vB5
Total protein (μg/ml)	120.52	452.47	573.74
HA conc. (μg/ml)	46.16	76.89	206.43
Total protein/HA	2.61	5.88	2.78

Figure S1. HA content of purified rRG56B-vB5 was analysed by densitometry.

8 µg of purified rRG56B-vB5 viruses were treated with PNGase F at 37°C for overnight. Each band was separated in 12% SDS-PAGE with 1X TG-SDS running buffer.

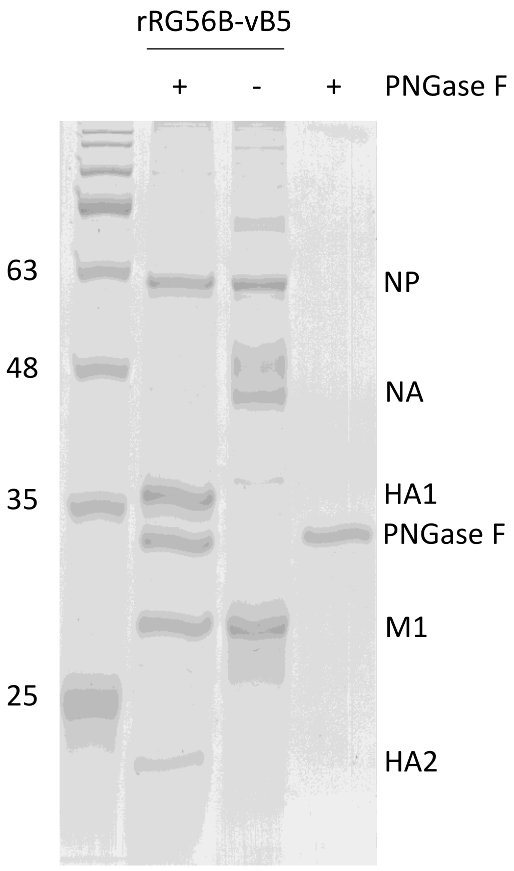

The percentage of HA: 35.98%

Figure S2. **Growth property** **of reassortant viruses in M199 medium in 6-well plates.**

Vero cells were cultivated in 6-well plates with serum-containing M199. The medium was replaced to serum-free M199 medium before infection. The MOI for the infection is 0.001. TPCK-trypsin was supplied, and the culture medium were sampled every day for TCID and HA assay. (a) The virus titre. (b) The HA titre. (c) The peak titre.

a

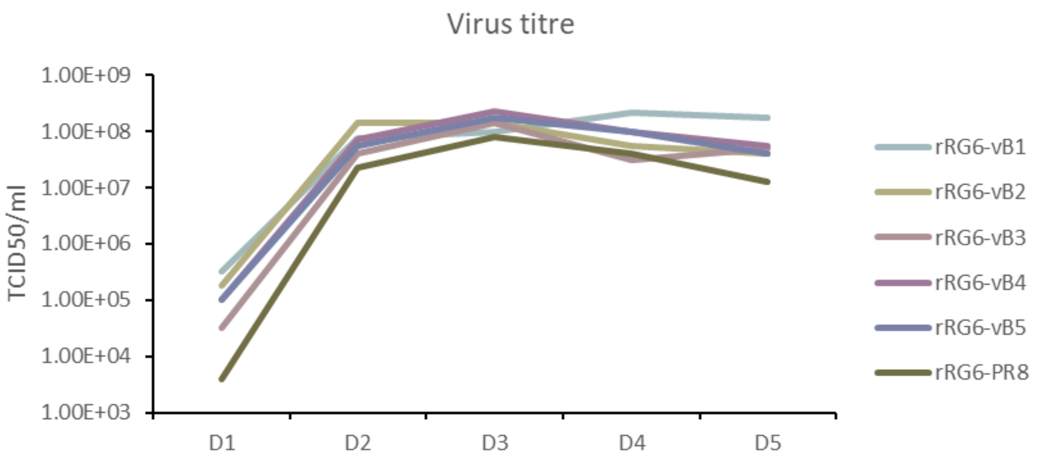

b

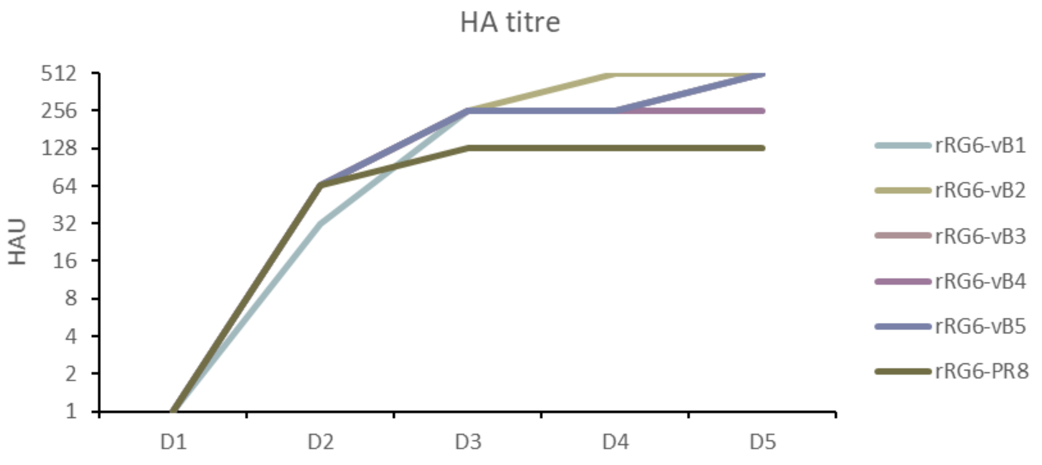

c

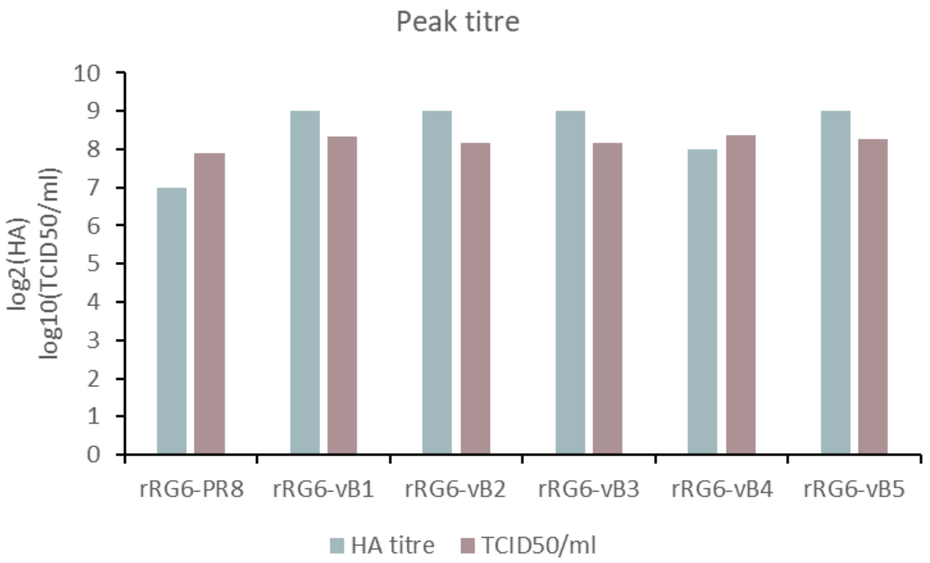

Peak titre
log2(HA)
log10(TCID50/ml)
10
9
8
7
6
5
4
3
2
1
0
rRG6-PR8
rRG6-vB1
rRG6-vB2
rRG6-vB3
rRG6-vB4
rRG6-vB5
HA titre
TCID50/ml

Figure S3. **Virus growth of rRG6-vB5 in MDCK cells.** MDCK cells were cultivated on Cytodex 1 microcarriers with OPTI-PRO SFM. Before infection, the culture medium was replaced to fresh one. The infection MOI was 0.0001. The samples collected every day were analysed for TCID and HA. (A) The HA titre (B) The virus titre (C) The peak HA titre.

A.

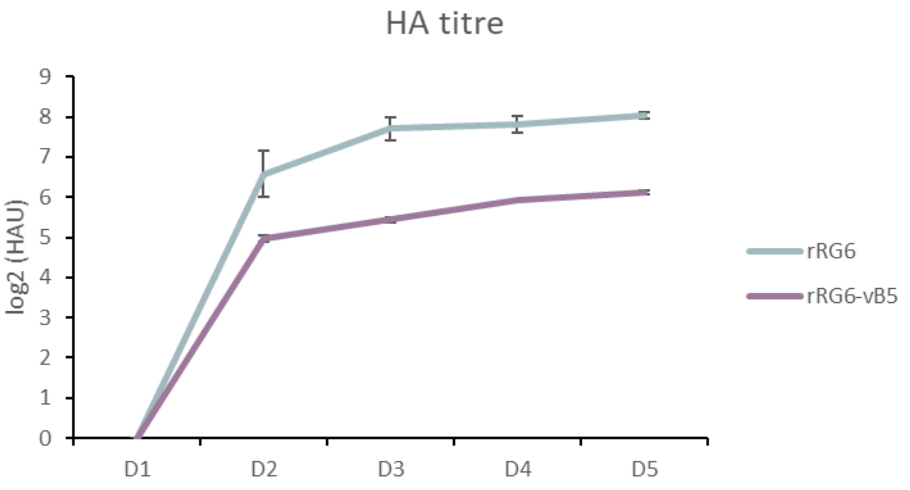

B.

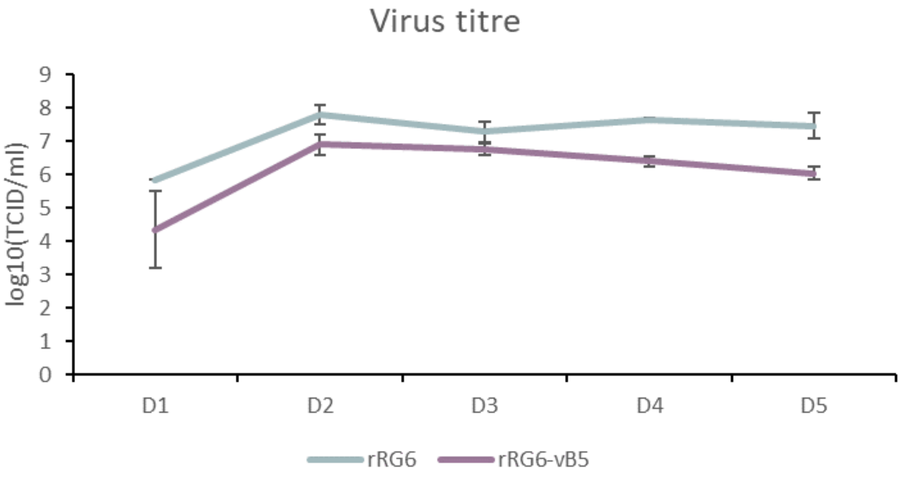

C.

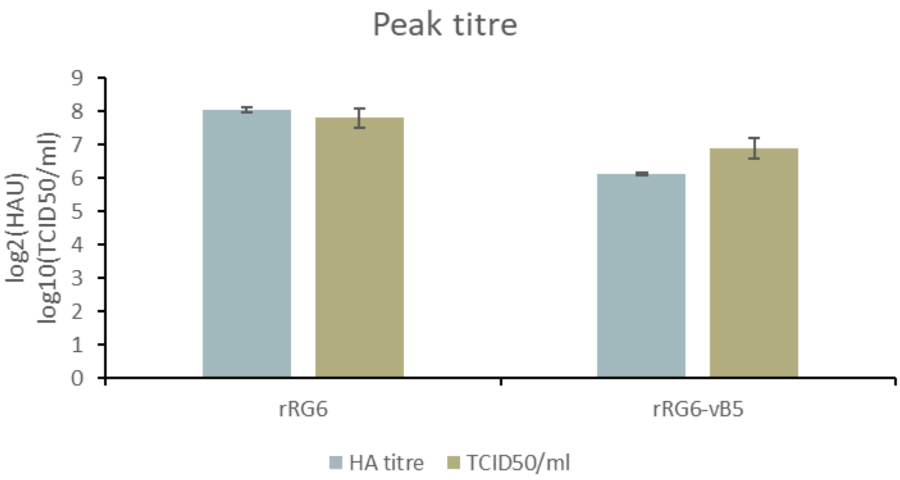

Peak titre
log2(HAU)
log10(TCID50/ml)
9
8
7
6
5
4
3
2
1
0
rRG6
rRG6-vB5
HA titre
TCID50/ml
